# A HPLC-based Method for Counting the Genome Copy Number of Cells Allows the Production of a High-quality Mock Community of Bacterial Cells

**DOI:** 10.1264/jsme2.ME24076

**Published:** 2025-05-10

**Authors:** Yoshifumi Ohyama, Takamasa Miura, Masataka Furukawa, Mamiko Shimamura, Yuki Asami, Atsushi Yamazoe, Yoshihito Uchino, Hiroko Kawasaki

**Affiliations:** 1 Biological Resource Center, National Institute of Technology and Evaluation (NBRC), 2–5–8 Kazusa-kamatari, Kisarazu, Chiba 292–0818, Japan

**Keywords:** mock communities, metagenome, high-throughput sequencing, control sample, HPLC

## Abstract

Improving the reliability of a metagenomic sequencing ana­lysis requires the use of control samples, known as mock communities. Therefore, mock communities must be prepared with high accuracy and reproducibility, which is particularly challenging for cellular mock communities. In the present study, we prepared a cellular mock community consisting of bacterial strains representative of the human and surrounding environmental microbiomes to demonstrate the suitability of a HPLC-based method that measures the genome number of cells. This method proved to be more accurate and reproducible for preparing cellular mock communities than traditional cell counting-based enumeration methods.

Microorganisms are ubiquitous and have a significant impact on the environment by shaping ecosystems to support various life forms. Understanding the role of microorganisms in their habitats has become crucial across various scientific fields. However, traditional culturing techniques have been limited in analyzing microbiota due to the huge‍ ‍number, complex compositions, and relationships of microbes as well as their uncultivability. In the past 20 years, the innovations of high-throughput sequencing (HTS) technology have overcome these issues (Arnold *et al.*, 2016). It is now possible to comprehensively analyze the genetic information of microbiota, *i.e.*, the metagenome, in their habitats (Nam *et al.*, 2023). However, the reliability of a metagenomic ana­lysis using HTS technology is still controversial due to the existence of different protocols and kits specialized for specific instruments and, more generally, due to the experimental biases introduced at each step of sample collection, DNA extraction, library preparation, HTS sequencing, and informatics ana­lysis (Costea *et al.*, 2017; Sinha *et al.*, 2017; Amos *et al.*, 2020; Tourlousse *et al.*, 2021, 2022; Mori *et al.*, 2023). This not only complicates the comparison of individual findings, but also leads to potentially erroneous conclusions (Hornung *et al.*, 2019).

To overcome these difficulties, the standardization of a metagenomic sequencing (MGS) ana­lysis is currently being promoted worldwide (Costea *et al.*, 2017; Tourlousse *et al.*, 2021, 2022). As an important essential material for standardization, experimental control samples adapted to a MGS ana­lysis have been developed (Sinha *et al.*, 2015; Allen-Vercoe *et al.*, 2019; Amos *et al.*, 2020; Tourlousse *et al.*, 2021, 2022). The use of experimental control samples is expected to measure the accuracy of a MGS ana­lysis at each step of the protocol, thereby ensuring the traceability of the data obtained and markedly increasing the reliability of the ana­lysis. Although several types of experimental control samples have been developed, mock communities are currently the most widely used (Allen-Vercoe *et al.*, 2019). Mock communities are mixtures of representative species of the microbiota of the target environment or a mixture of their extracted DNA. Ideally, mock communities need to be‍ ‍prepared according to a specific design that fixes the compositional ratios and biological masses of separately cultured microbes, which establishes a reference value, or “ground truth”, for experiments (Tourlousse *et al.*, 2021, 2022). The use of mock communities allows for the verification of experimental accuracy and identification of experimental bias. In addition, the advantage of mock communities is that they may be repeatedly produced without any quantitative limitations; therefore, they may be used by microbiome researchers without any time-related constraints, which fulfils the essential feature of standard samples. However, to the best of our knowledge, there have been no studies that have detailed how to repeatedly produce mock communities according to the planned design. The repeated production of cellular mock communities (hereafter referred to as the cell mock) of consistent quality is challenging because cultured cells exhibit variations in growth rates, morphologies, and other characteristics.

In our collaborative research project on the standardization of metagenomic methods (Tourlousse *et al.*, 2021, 2022), we utilized a HPLC-based method to quantify bacterial genomic DNA by measuring adenine from depurinated genomes (de Bruin and Birnboim, 2016) in order to produce a cell mock for the human gut microbiome. This method was selected for its potential to provide more accurate and reproducible quantification than traditional cell counting techniques. Although we demonstrated that this cell mock had sufficient qualities to validate metagenomic ana­lyses of human gut microbes (Tourlousse *et al.*, 2022), we did not fully examine how accurately and reproducibly the cell mock may be produced across a wider range of bacterial species. In the present study, we aimed to extend our previous work by investigating whether the HPLC-based quantitative method produces a high-quality cell mock with new strain combinations, representing a broader range of microbiomes living in and around us. Our goal was to establish this method as a robust alternative to conventional cell counting, achieving high accuracy and reproducibility across various bacterial types, thereby enhancing the reliability and comparability of metagenomic ana­lyses in different research contexts.

We used three different bacterial counting methods: microscopy, flow cytometry and a HPLC-based method (de Bruin and Birnboim, 2016), in which the selected bacterial strains were grown to the late logarithmic and early stationary phase. Details on the medium and culture conditions are shown in Table S1. These cultured bacteria were collected by centrifugation, washed once with PBS, and resuspended in PBS with a volume equal to the culture volume (this resuspension is hereafter referred as the initial cell resuspension). In preparation for microscopy, the initial cell resuspension was diluted to 1 absorbance unit at 600‍ ‍nm, further diluted 1000-fold, and mixed with a 1/1000 volume of SYTO^TM^ 9 dye (ThermoFisher Scientific). The mixture was incubated at room temperature for 15‍ ‍min for cell staining. Stained cells were trapped by filtration through a filter with a mesh size of 0.45‍ ‍μm and the filter was supplied for fluorescent microscopic observations (Nikon). At least 30 images were captured for each strain and an image ana­lysis for cell counting was performed using Fiji (Schindelin *et al.*, 2012). In the flow cytometric ana­lysis, the initial cell resuspension was diluted 1000-fold and dilutions were stained with SYTO^TM^ 9 dye as described above. Stained dilutions were mixed with a 1/100 volume of *ϕ* 6‍ ‍μm microsphere standard beads (a component of the Bacteria Counting Kit; ThermoFisher Scientific) and the mixtures were measured using a flow cytometer (CytoFLEX; Beckman Coulter). The HPLC-based method (de Bruin and Birnboim, 2016) was slightly modified to calculate the number of genomes of bacterial cells in the initial cell resuspension, as described by Tourlousse *et al.* (2021). The genome size and GC content of each bacterium used for this calculation are listed in Table 1. All of the above enumeration methods were performed three times independently. Along with cell or genome number counting, a portion of the initial cell resuspension was centrifuged again, and the resulting pellets were suspended in an arbitrary volume of PBS containing 15% (v/v) glycerol to condense and store them at –80°C for later use. To prepare the cell mock, the concentration of each PBS-glycerol stock was calculated with the values and dilution/condensation ratio of each enumeration method, the equal number of cells/genomes were mixed at 4×10^10^ mL^–1^ and 100 aliquots (100‍ ‍μL) were made per batch. Regarding DNA mock communities (hereafter referred to as the DNA mock), high-mole­cular-weight DNA from each strain was extracted using the MagAttract HMW DNA kit (QIAGEN). The concentration of purified DNA was measured with the Quant-iT^TM^ PicoGreen^TM^ dsDNA Assay Kit (ThermoFisher Scientific) and the genomic molar concentration of purified DNA was calculated using the genome size from Table 1 and the average mole­cular weight of duplex DNA, 660. Purified DNA containing the equimolar genomes of each bacterium was mixed, and the final concentration of the mixed solution was adjusted to 50‍ ‍ng μL^–1^. Fifty aliquots (30‍ ‍μL) of the mixture were prepared per batch as the DNA mock. Two orthogonal methods, a shotgun metagenomic ana­lysis and a type of quantitative PCR, droplet digital PCR (ddPCR) (Bio-Rad), were used to assess strain-wise abundance in the cell mock and DNA mock. To extract DNA from the cell mock, we used the ISOSPIN Fecal DNA kit (NIPPON GENE) as described in the Japan Microbiome Consortium (JMBC) standard operating procedures (SOP) for the metagenomic ana­lysis of human feces (hereafter referred to as JMBC SOP; https://jmbc.life/sop/index.html) (Tourlousse *et al.*, 2021, 2022). In the shotgun metagenomic ana­lysis, a sequencing library was prepared with the ThruPLEX DNA-Seq Kit (TaKaRa Bio) as described in JMBC SOP (Tourlousse *et al.*, 2021, 2022) and sequencing was performed with the MiSeq Reagent Kit v2 (300 cycles) and MiSeq system (Illumina). Sequencing reads were preprocessed by fastp (v0.20.0; Chen *et al.*, 2018) with the following command line: --trim_front1 5 --trim_front2 5 --trim_tail1 1 --trim_tail2 1 --cut_right --cut_right_window_size 4 --cut_right_mean_quality 18 --trim_poly_x --poly_x_min_len 10 --n_base_limit 0 --low_complexity_filter --length_required 75, and preprocessed reads were used to quantify the relative abundance of the cell and DNA mocks using the kallisto (v0.46.1; Bray *et al.*, 2016) quantification algorithm (kallisto quant) with the default command line and reference genome sequences consisting of only 15 bacterial strains, as described by Tourlousse *et al.* (2021, 2022). In ddPCR measurements, 30‍ ‍ng of DNA from the cell or DNA mock was digested with the restriction enzyme *Rsa*I and diluted to 30 pg μL^–1^ with pure water. The diluted solution was used for ddPCR as follows. Twenty microliters of the PCR reaction solution containing 210 pg of *Rsa*I-digested DNA, 1‍ ‍mM FITC, 1× PrimeTime^TM^ qPCR Probe Assays (Integrated DNA Technologies; the designed sequences of primers and probes for each strain are listed in Table S2), and 1× ddPCR^TM^ Supermix for Probes (Bio-Rad) were supplied for droplet generation according to the ddPCR system instructions. The micellar PCR solution was subjected to the following thermal cycling program: at 98°C for 2‍ ‍min, 40 cycles at 98°C for 2‍ ‍min-70°C for 2‍ ‍min, and at 70°C for 2‍ ‍min. The PCR solution was then subjected to the measurement of fluorescently active droplets according to the ddPCR system instructions. The number of fluorescent droplets of each bacterial strain obtained was used to calculate the relative abundance of each bacterium in the cell and DNA mocks. The above shotgun metagenomic ana­lysis and ddPCR assays were performed in triplicate. The basic statistics obtained from the variabilities of relative abundance were used to calculate the coefficient of variation (CV). Absolute fold differences (AFD) were calculated as described by Tourlousse *et al.* (2021) using the ratio between the relative and theoretical abundance of each bacterium (theoretical abundance in equally mixed cell and DNA mocks, 6.7%). Welch’s *t*-test was performed to establish whether the variabilities of CV and AFD showed significant differences.

We aimed to provide a versatile control sample for a metagenomic ana­lysis for researchers interested in the microbiome associated with daily life and, thus, selected human symbionts and bacteria inhabiting our surrounding environment as the components of new mock communities. To confirm the issues associated with a metagenomic ana­lysis, we selected Gram-positive and -negative bacteria as a marker of their cell wall strength causing experimental bias in the DNA extraction step, and they were incorporated into the mock communities with a near-balanced composition. In addition, bacteria with a wide range of GC contents, also a contributor to experimental bias, as well as genome sizes and 16S rRNA gene copy numbers were incorporated. Moreover, only bacterial strains below biosafety level 2 were selected to facilitate the handling of mock communities in each laboratory. All 15 bacterial strains and genome information are listed in Table 1 and Gram stain and isolation source information in Table S3.

We aimed to produce a compositionally accurate cell mock, focusing on the method described by de Bruin and Birnboim (2016), in which the total amount of bacterial genomic DNA was quantified as follows. Target bacterial cells were treated with strong acid to depurinate genomic DNA and released adenine was detected by HPLC. The amount of adenine was converted to the amount of DNA by using GC content and mole­cular weight of dNMPs as described in their study (de Bruin and Birnboim, 2016). Since the amount of DNA may be converted to the number of genomic DNA by dividing with the mass of a single particle of the genome, and the number of genomic DNA may then be used as an approximate number of bacterial cells, we confirmed whether their method (hereafter referred to as adenine-HPLC) was able to count bacterial cells.

We used *Escherichia coli*, one of the 15 strains of mock communities whose cells are discrete (Fig. S1) and suitable for conventional cell counting by microscopy and flow cytometry. We compared the numbers of *E. coli* by measuring with conventional methods and adenine-HPLC (Fig. S2). The results obtained showed that adenine-HPLC measurements were ~1.4- and ~1.1-fold higher than microscopy and flow cytometry, respectively. As described above, the value of the adenine-HPLC measurement is the number of genomic DNA, which is different from the number of cells measured by the conventional methods. Since the bacterial cultures used for these measurements were from the late logarithmic growth phase and contained cells undergoing DNA replication, it was reasonable that the genome counts obtained by adenine-HPLC were higher than those obtained by the conventional methods. Therefore, we consider adenine-HPLC to accurately measure the number of genomes and decided to generate a new cell mock using this method. We then measured and compared the numbers of 15 constituent strains, including *E. coli*, using the above three methods (Fig. 1). The results obtained showed that adenine-HPLC gave higher counts than the other methods for all 15 strains, which is consistent with the number of genomes being higher than the number of cells.

We then prepared three batches of cell mocks by mixing equal amounts of cells/genomes from the 15 bacterial strains based on flow cytometry and adenine-HPLC measurements. It is important to note that we excluded microscopy for these preparations because microscopic observations are a fully manual operation that requires technical skill by the operator, while the other methods are semi-automated and require a shorter operation time. To confirm the accuracy of mixing, the DNA of the cell mock was extracted according to the‍ ‍DNA extraction method described in JMBC SOP (Tourlousse *et al.*, 2021, 2022), which has been shown to extract DNA from Gram-positive and -negative bacteria with‍ ‍almost equal efficiency. Extracted DNA was then used‍ ‍to assess the compositional ratio of the cell mock with two orthogonally different assessing methods, ddPCR and the shotgun sequencing ana­lysis. In addition, to clarify the experimental accuracy of the two assessing methods, the DNA mock was also prepared by mixing equal amounts of DNA extracted from each of the 15 strains and used as experimental controls. The resulting compositions are shown in Fig. 2. The compositions obtained by ddPCR and shotgun sequencing were very similar to each other (Fig. 2A). We evaluated the quality of the cell and DNA mocks using two criteria. The reproducibility of the preparation of the mocks was compared between the three batches by calculating variability in the compositional ratio of each strain (Fig. 2B). We found that the DNA mock had the highest reproducibility and showed no significant differences in variabilities between shotgun sequencing and ddPCR; therefore, we considered these two assays to perform measurements with similar accuracy. On the other hand, reproducibility for the cell mock was different between the preparation methods, *i.e.*, flow cytometry and adenine-HPLC, and it became clear that adenine-HPLC, with less variation in reproducibility, was more accurate as a method for preparing the cell mock. As a second criterion, closeness to the expected value of the composition was calculated. The ratio between the expected composition and measured compositions of each strain was compared in the cell and DNA mocks (Fig. 2C and D). The results obtained showed that the composition of the cell mock from adenine-HPLC was significantly closer to the expected value than that from flow cytometry in the both shotgun and ddPCR measurements, indicating that the cell mock was prepared more accurately by adenine-HPLC in this criterion. Notably, the superiority of adenine-HPLC for the qualities of the cell mock over microscopy using a different set of strains in our preliminary observations was also confirmed.

We then compared the compositions of the 15 constituent bacteria individually (Fig. S3). In the cell mock prepared by‍ ‍flow cytometry, Gram-negative bacteria with relatively discrete morphologies were less abundant than expected, with the exception of *Acinetobacter radioresistens*, and this‍ ‍may be mainly due to the undercounting of bacteria, such as *Cutibacterium acnes* subsp. *acnes* exhibiting strong aggregation (Fig. S1) and *Lactobacillus delbrueckii* with a filamentous morphology, due to difficulties distinguishing cell-cell boundaries (Fig. S1). On the other hand, in the cell‍ ‍mock prepared by adenine-HPLC, the strains with small‍ ‍genome sizes, except for *C. acnes* subsp. *acnes*, were‍ ‍less abundant, particularly in *Bifidobacterium pseudocatenulatum* (Fig. S3), indicating that adenine-HPLC was more likely to overestimate the amount of these strains. However, it is important to note that this result was not necessarily reproduced in the other strains with smaller genomes exami­ned outside of this study (Tourlousse *et al.*, 2022, and data not shown). In any case, Fig. S3 shows that even though adenine-HPLC still has room for improvement in terms of quantitativeness in the future, it is also clear from the following points that adenine-HPLC will continue to be a useful quantification method in microbiology in the future. In the bioinformatics ana­lysis of microorganisms based on HTS technology, the basic unit of biomass is not the conventional cell, but the sequence of the genome or gene, such as amplicons, which is in a completely consistent or proportional relationship with the basic unit of adenine-HPLC. Therefore, adenine-HPLC may be regarded as a quantitative method that is extremely compatible with a microbial bioinformatics ana­lysis.

In the present study, we developed new mock communities suitable for human-related microbiome research and successfully produced a more accurate and reproducible cell mock than that produced by the conventional method using adenine-HPLC. We confirmed the quantitative reproducibility of this method in all of the several dozens of bacterial strains studied so far, regardless of the handling operators (Tourlousse *et al.*, 2022, and data not shown). Therefore, this method will allow the customization of high-quality cell mock production by freely selecting bacterial strains to suit the purpose of various microbiome research applications, and will lead to improvements in the quality of the cell mock and the reliability of the MGS ana­lysis. Mock communities with equivalent qualities in the present study are currently available from our organization, NBRC, under the names Cell-Mock-002 and DNA-Mock-002 (https://www.nite.go.jp/nbrc/industry/microbiome/cocktail.html).

All sequencing data have been uploaded to the DDBJ Sequence Read Archive under BioProject PRJDB18400.

## Citation

Ohyama, Y., Miura, T., Furukawa, M., Shimamura, M., Asami, Y., Yamazoe, A., et al. (2025) A HPLC-based Method for Counting the Genome Copy Number of Cells Allows the Production of a High-quality Mock Community of Bacterial Cells. *Microbes Environ ***40**: ME24076.

https://doi.org/10.1264/jsme2.ME24076

## Supplementary Material

Supplementary Material 1

Supplementary Material 2

## Figures and Tables

**Fig. 1.**
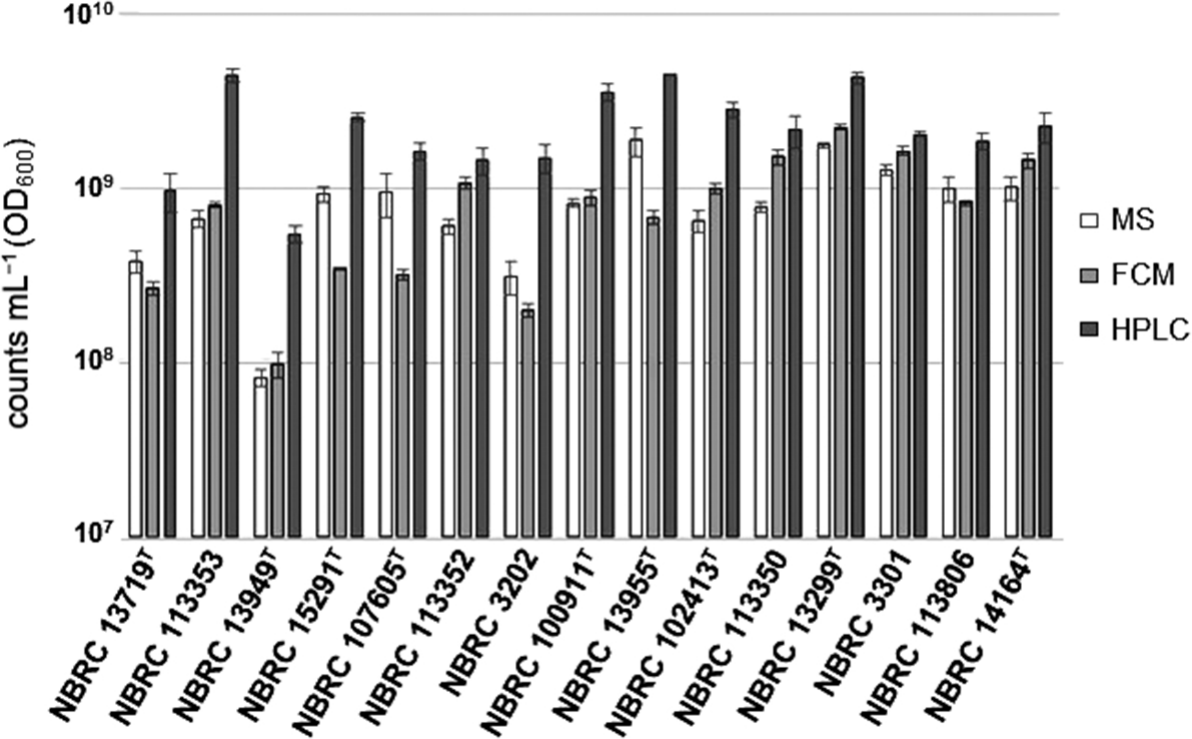
Enumeration of bacterial components of mock communities by three independent methods. The counts of each method are expressed as adjusted to 1 unit of OD_600_ (*n*=3±standard deviations). The three methods used the same culture of each strain, grown independently between replicates. MS: microscope, FCM: flow cytometer.

**Fig. 2.**
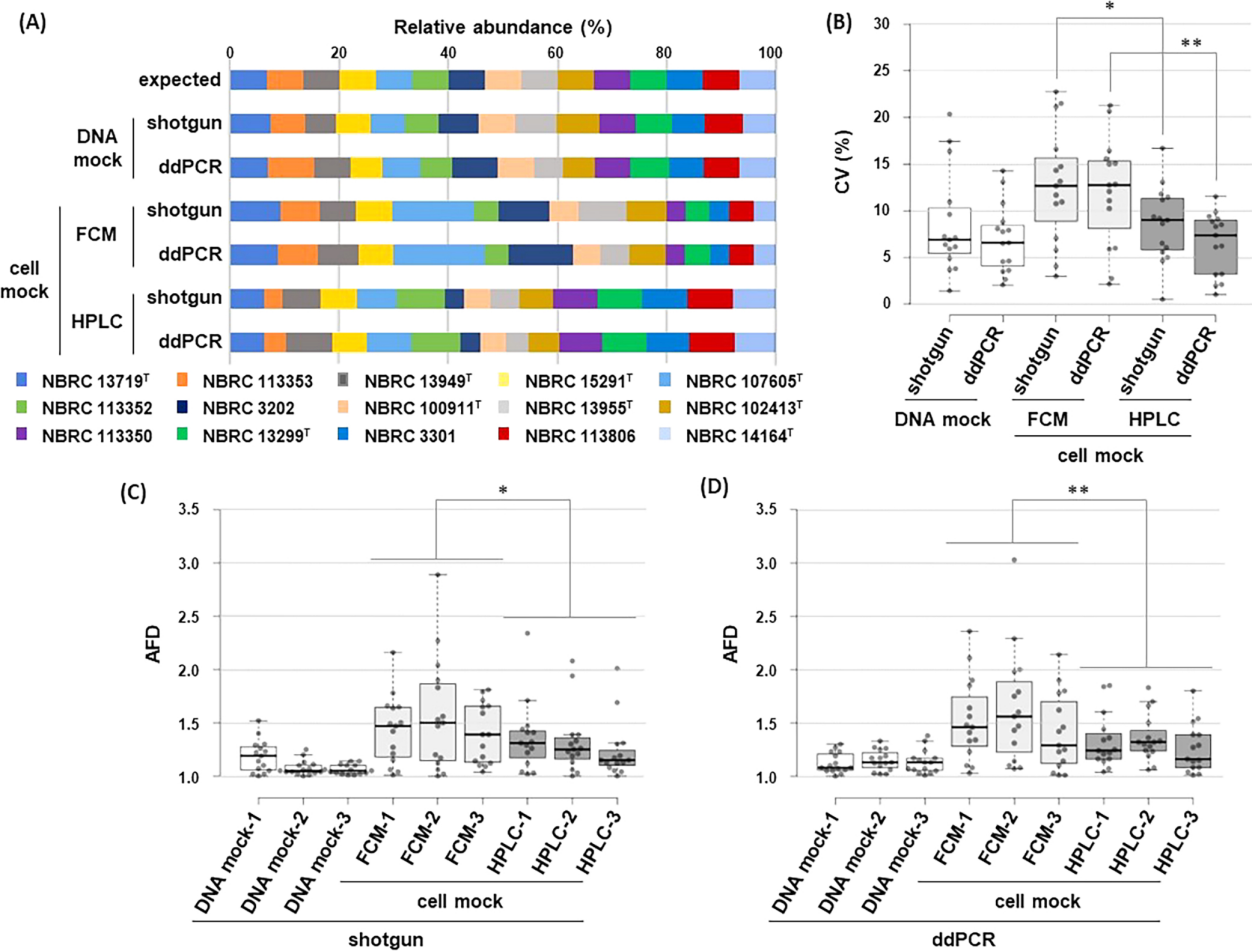
Composition of the DNA mock and cell mock. (A) The relative abundance of each strain in the DNA mock and cell mock assessed by shotgun sequencing and ddPCR is shown. Values are the mean of three batches. (B) The variabilities of the relative abundance of each strain in the three batches of the DNA mock and cell mock measured by shotgun sequencing and ddPCR were calculated as the coefficient of variation (CV) and presented as boxplots. Boxes ranged in the 25th to 75th percentiles with the median, horizontal thick line, and whiskers extending to the largest and smallest values at most 1.5× the interquartile range from the top and bottom sides of the boxes. The significance of differences in some data was analyzed by Welch’s *t*-test (*P<0.05, **P<0.01) (C), (D) The variabilities of the ratios between the expected composition and the composition per each strain measured by shotgun sequencing (C) and ddPCR (D) were expressed as absolute fold differences (AFD). AFD were calculated for each strain among each of the three batches of the DNA mock and cell mock and presented as boxplots. Boxplots are indicated as in (B) (*P<0.05, **P<0.01). Independently cultured cells were used to prepare each batch (A–D).

**Table 1.** Information on 15 strains mixed in mock communities.

Strain	Genome size (bp)	GC content (%)	16S copies	Accession number
*Bacillus subtilis* NBRC 13719^T^	4,295,305	43.3	10	AP019714, AP019715
*Bifidobacterium pseudocatenulatum* NBRC 113353	2,277,780	56.4	5	BJLC00000000
*Clostridium butyricum* NBRC 13949^T^	4,705,096	28.8	11	AP019716–AP019719
*Corynebacterium striatum* NBRC 15291^T^	3,113,921	59.1	4	BJLD00000000
*Cutibacterium acnes* subsp. *acnes* NBRC 107605^T^	2,494,738	60.0	3	AP019723
*Enterocloster clostridioformis* NBRC 113352	5,687,315	48.9	5	BJLB00000000
*Lactobacillus delbrueckii* NBRC 3202	1,910,306	50.1	8	AP019750
*Staphylococcus epidermidis* NBRC 100911^T^	2,427,041	32.3	6	AP019721, AP019722
*Streptococcus mutans* NBRC 13955^T^	2,018,796	36.9	5	AP019720
*Acinetobacter radioresistens* NBRC 102413^T^	3,433,938	41.4	6	AP019740–AP019748
*Bacteroides uniformis* NBRC 113350	4,989,532	46.2	4	AP019724–AP019728
*Comamonas terrigena* NBRC 13299^T^	4,673,011	65.0	7	AP019749
*Escherichia coli* NBRC 3301	4,755,096	50.8	7	CP048439, CP048440
*Parabacteroides distasonis* NBRC 113806	5,179,960	45.0	7	AP019729
*Pseudomonas putida* NBRC 14164^T^	6,156,701	62.3	7	AP013070
