# Supplementary material for: A HPLC-based Method for Counting the Genome Copy Number of Cells Allows the Production of a High-quality Mock Community of Bacterial Cells
Source: Microbes Environ. 2025 May 10;40(2):ME24076. doi: 10.1264/jsme2.ME24076 (PMC12213061; doi:10.1264/jsme2.ME24076)
Supplement: Supplementary file 1 — Supplementary Material 1 [file 40_24076_s1.pdf]

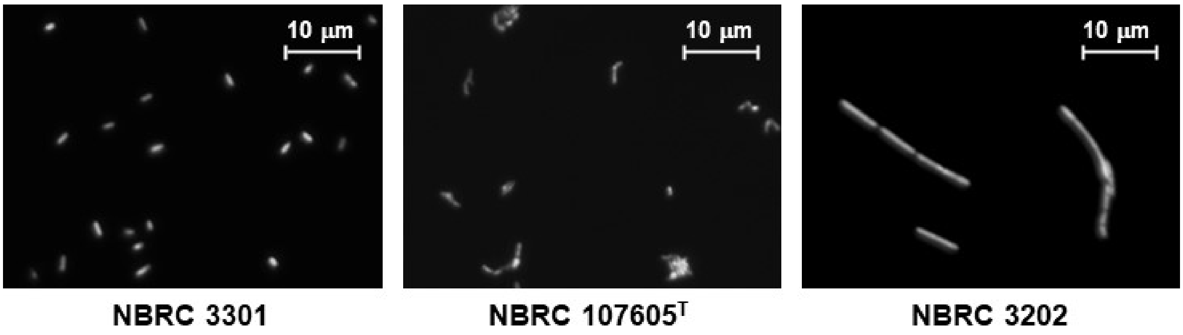

Fig. S1. The three morphologically representative strains, *E. coli* (NBRC 3301), *C. acnes* subsp. *acnes* (NBRC 107605^T) and *L. delbrueckii* (NBRC 3202), are shown. Each strain was stained with SYTO9^{TM} fluorescent dye (ThermoFisher Scientific), collected using mesh filter, and the filter was mounted on a fluorescence microscope (Nikon) to capture the cell images.

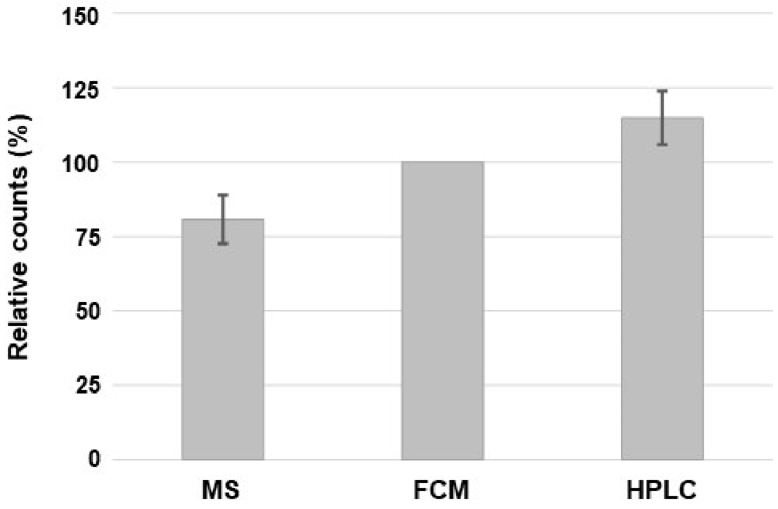

Fig. S2. The enumeration of *E. coli* by microscope (MS), flow cytometer (FCM) and adenine-HPLC (HPLC). The MS and HPLC counts are expressed as relative values to FCM counts (n =3 ± standard deviations).

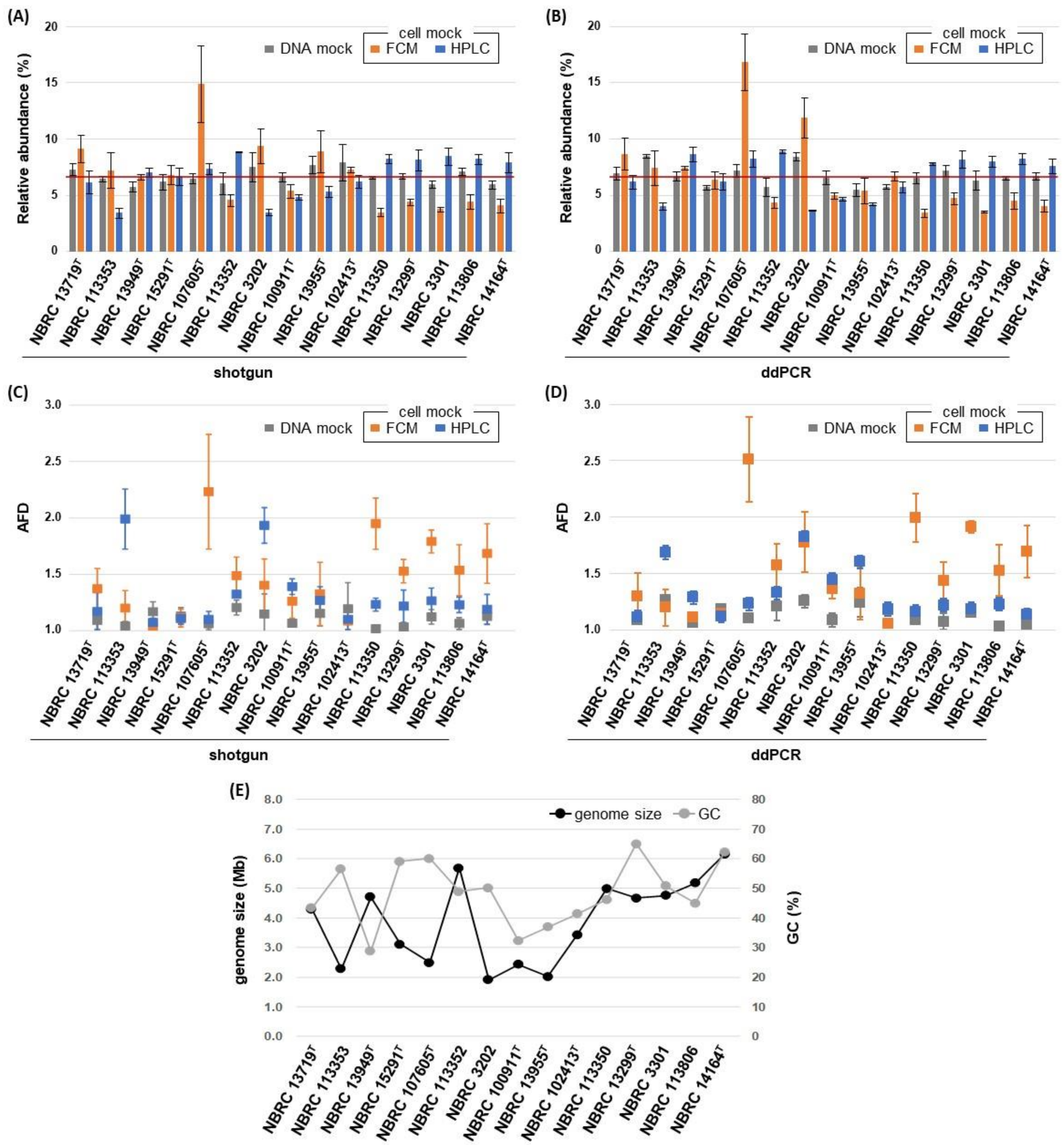

Fig. S3. (A), (B) Relative abundances of 15 constituent strains in DNA mock and cell mock as determined by shotgun sequencing (A) and ddPCR (B) are shown according to the preparation methods (n =3 ± standard deviations). The red line indicates the expected abundance, 6.7%. (C), (D) AFD between each constituent strain's composition and expected composition in DNA mock and cell mock as determined by shotgun sequencing (C) and ddPCR (D) are shown according to the preparation methods (n =3 ± standard deviations). (E) Genome size and GC content of 15 constituent strains in the DNA mock and cell mock.

Supplemental References

1. Bernard, K.A., and Funke, G. (2015) Corynebacterium In *Bergey's Manual of Systematics of Archaea and Bacteria*. Whitman, W.B., Aharal, D.R., Christensen, H., Chuvochina, M., Dedysh, S., Gasparich, G.E., et al. (eds) Hoboken, NJ: John Wiley & Sons, Inc. 1-70.

2. Biavati, B., and Mattarelli, P. (2015) Bifidobacterium In *Bergey's Manual of Systematics of Archaea and Bacteria*. Whitman, W.B., Aharal, D.R., Christensen, H., Chuvochina, M., Dedysh, S., Gasparich, G.E., et al. (eds) Hoboken, NJ: John Wiley & Sons, Inc. 1-57.

3. Hammes, W.P., and Hertel, C. (2015) Lactobacillus In *Bergey's Manual of Systematics of Archaea and Bacteria*. Whitman, W.B., Aharal, D.R., Christensen, H., Chuvochina, M., Dedysh, S., Gasparich, G.E., et al. (eds) Hoboken, NJ: John Wiley & Sons, Inc. 1-76.

4. Logan, N.A., and DeVos, P. (2015) Bacillus In *Bergey's Manual of Systematics of Archaea and Bacteria*. Whitman, W.B., Aharal, D.R., Christensen, H., Chuvochina, M., Dedysh, S., Gasparich, G.E., et al. (eds) Hoboken, NJ: John Wiley & Sons, Inc. 1-164.

5. Nemec, A. (2022) Acinetobacter In *Bergey's Manual of Systematics of Archaea and Bacteria*. Whitman, W.B., Aharal, D.R., Christensen, H., Chuvochina, M., Dedysh, S., Gasparich, G.E., et al. (eds) Hoboken, NJ: John Wiley & Sons, Inc. 1-78.

6. Palleroni, N.J. (2015) Pseudomonas In *Bergey's Manual of Systematics of Archaea and Bacteria*. Whitman, W.B., Aharal, D.R., Christensen, H., Chuvochina, M., Dedysh, S., Gasparich, G.E., et al. (eds) Hoboken, NJ: John Wiley & Sons, Inc. 1-105.

7. Patrick, S., and McDowell, A. (2015) Propionibacterium In *Bergey's Manual of*

Systematics of Archaea and Bacteria. Whitman, W.B., Aharal, D.R., Christensen, H., Chuvochina, M., Dedysh, S., Gasparich, G.E., et al. (eds) Hoboken, NJ: John Wiley & Sons, Inc. 1-29.

8. Rainey, F.A., Hollen B.J., and Small, A.M. (2015) Clostridium In *Bergey's Manual of Systematics of Archaea and Bacteria*. Whitman, W.B., Aharal, D.R., Christensen, H., Chuvochina, M., Dedysh, S., Gasparich, G.E., et al. (eds) Hoboken, NJ: John Wiley & Sons, Inc. 1-122.

9. Scheutz, F., and Strockbine, N.A. (2015) Escherichia In *Bergey's Manual of Systematics of Archaea and Bacteria*. Whitman, W.B., Aharal, D.R., Christensen, H., Chuvochina, M., Dedysh, S., Gasparich, G.E., et al. (eds) Hoboken, NJ: John Wiley & Sons, Inc. 1-49.

10. Schleifer, K., and Bell, J.A. (2015) Staphylococcus In *Bergey's Manual of Systematics of Archaea and Bacteria*. Whitman, W.B., Aharal, D.R., Christensen, H., Chuvochina, M., Dedysh, S., Gasparich, G.E., et al. (eds) Hoboken, NJ: John Wiley & Sons, Inc. 1-43.

11. Song, Y., Liu, C., and Finegold, S.M. (2015) Bacteroides In *Bergey's Manual of Systematics of Archaea and Bacteria*. Whitman, W.B., Aharal, D.R., Christensen, H., Chuvochina, M., Dedysh, S., Gasparich, G.E., et al. (eds) Hoboken, NJ: John Wiley & Sons, Inc. 1-24.

12. Whiley, R.A., and Hardie, J.M. (2015) Streptococcus In *Bergey's Manual of Systematics of Archaea and Bacteria*. Whitman, W.B., Aharal, D.R., Christensen, H., Chuvochina, M., Dedysh, S., Gasparich, G.E., et al. (eds) Hoboken, NJ: John Wiley & Sons, Inc. 1-86.

13. Willems, A., and Gillis, M. (2015) Comamonas In *Bergey's Manual of Systematics of Archaea and Bacteria*. Whitman, W.B., Aharal, D.R., Christensen, H., Chuvochina, M., Dedysh, S., Gasparich, G.E., et al. (eds) Hoboken, NJ: John Wiley & Sons, Inc. 1-17.
