# Supplementary material for: A HPLC-based Method for Counting the Genome Copy Number of Cells Allows the Production of a High-quality Mock Community of Bacterial Cells
Source: Microbes Environ. 2025 May 10;40(2):ME24076. doi: 10.1264/jsme2.ME24076 (PMC12213061; doi:10.1264/jsme2.ME24076)
Supplement: Supplementary file 2 — Supplementary Material 2 [file 40_24076_s2.pdf]

Table S1. Culture conditions of the 15 strains mixed in mock communities

Strain	[a]BSL	[b]medium	temperature (°C)	time (h)	culturing conditions
Bacillus subtilis NBRC 13719[T]	1	702	30	24	aerobic (180 rpm shaking)
Bifidobacterium pseudocatenulatum NBRC 113353	1	1509	37	24	anaerobic (N_2 atmosphere)
Clostridium butyricum NBRC 13949[T]	1	1509	37	24	anaerobic (N_2 atmosphere)
Corynebacterium striatum NBRC 15291[T]	1*	702	30	24	aerobic (180 rpm shaking)
Cutibacterium acnes subsp. *acnes* NBRC 107605[T]	1*	1509 + 1% (w/v) glucose	37	48	anaerobic (N_2 atmosphere)
Enterocloster clostridioformis NBRC 113352	1*	1509	37	24	anaerobic (N_2 atmosphere)
Lactobacillus delbrueckii NBRC 3202	1	804	30	32	anaerobic (N_2 atmosphere)
Staphylococcus epidermidis NBRC 100911[T]	1*	702	30	24	aerobic (180 rpm shaking)
Streptococcus mutans NBRC 13955[T]	1*	1509	37	24	anaerobic (N_2 atmosphere)
Acinetobacter radioresistens NBRC 102413[T]	1*	702	30	24	aerobic (180 rpm shaking)
Bacteroides uniformis NBRC 113350	1*	1509	37	24	anaerobic (N_2 atmosphere)
Comamonas terrigena NBRC 13299[T]	1*	702	30	24	aerobic (180 rpm shaking)
Escherichia coli NBRC 3301	1	702	30	24	aerobic (180 rpm shaking)
Parabacteroides distasonis NBRC 113806	1*	1509	37	24	anaerobic (N_2 atmosphere)
Pseudomonas putida NBRC 14164[T]	1*	702	30	24	aerobic (180 rpm shaking)

(a) BSL1* is a subclass of BSL1, which defines the bacteria that has been isolated from humans and can cause opportunistic infections in people with compromised immune systems.

(b) medium 702: https://www.nite.go.jp/nbrc/catalogue/NBRCMediumDetailServlet?NO=702

medium 804: https://www.nite.go.jp/nbrc/catalogue/NBRCMediumDetailServlet?NO=804

medium 1509: https://www.nite.go.jp/nbrc/catalogue/NBRCMediumDetailServlet?NO=001509

Table S2. Sequences of probes and primers for droplet digital PCR

Strain	*probe (5' -> 3')	primer (5' -> 3')
Bacillus subtilis NBRC 13719^{T}	TGGCGGCAAGAGCAAGAATGGCTGCG	GGCGATGGAAACATTTATGCTGGA<br>CTCTTACGGCGTGTTAGTTCACG
Bifidobacterium pseudocatenulatum NBRC 113353	CCGATGCCGATGTGGATGGCGCGC	GAACAAAGTCCGCTACCACAAGG<br>GAACAGCGTCAGATTCAGGGTTG
Clostridium butyricum NBRC 13949^{T}	ATTCTGCCGGCGGCTCAGCT	TCAACATTGCCTGGGAAATTAGC<br>TGCTTGGAACTTTCTATTTCTGCC
Corynebacterium striatum NBRC 15291^{T}	TGGTCCGAGCGAGGAAAAGCCGGCA	TGACATTGACGTTGACGCTGAAG<br>TGGAGGTCTTGGTCTTGTTGAGG
Cutibacterium acnes subsp. *acnes* NBRC 107605^{T}	CCGGCGGTAAGTTCGGCAACGGCGG	CTTTGGTACTGACCAAGCTGCAC<br>GACCGCTAGAGCATTAACAACCG
Enterocloster clostridioformis NBRC 113352	TCCGCAGGCGGTTCCGCAAA	CTGACTGCTCTGACAAAAATCCG<br>CAGAGGAAGTATGGCCTGAGTG
Lactobacillus delbrueckii NBRC 3202	TGCCGCTAAGCGCGCCCGGG	GATCGTTGAAAAGGGGCAACTGG<br>CGGGTCATTTGAAGTGTTGTCGG
Staphylococcus epidermidis NBRC 100911^{T}	TCACGCGCACGTGTGGCTGCCA	TGAGAATCCATCTGTAGGACGCAT<br>CGTCTAATGCTGATTTACGACGC
Streptococcus mutans NBRC 13955^{T}	TCATGCGTCCGGTGCTTGAAGCAGGCT	GACTGATGCTGATGTTGATGGGG<br>CATAAATAGGCGGTTGCGCGATA
Acinetobacter radioresistens NBRC 102413^{T}	CGGCAGGCGGTTCTGCCAAGCAGGG	GGAAAAAGATCCCGCCTTGTCTG<br>ATCGCCTGCATTTTACGGTTACG
Bacteroides uniformis NBRC 113350	TGTGCGCTACGTGGACTCCTCAC	AGAGGGAAATGCCAAGAAGGAAA<br>TACATCGTTCATGAAGTGTTCGC
Comamonas terrigena NBRC 13299^{T}	CGCGCCGCCATGACGCGCGT	TGTGCTTCACCAACAACATTCCC<br>GATTTCGACCTTGGCCTTCTTGG
Escherichia coli NBRC 3301	TCGACGAAGCGCTCGCGGGTCACT	TAAGCGCCCGGGTATGTATATCG<br>CAGAGTTATCGGCGTGAATGGTG
Parabacteroides distasonis NBRC 113806	CCGACGACGGCCGTGGTATCCCCG	ATGAGGACAACTCGATCACGGTT<br>CAGCACCGTCAATACTACCTCCA
Pseudomonas putida NBRC 14164^{T}	TGGACGAGTCGGCCCCAGCGGT	AGGAGCAGTACATCAAGGACGAC<br>GGAATTCGTTCACCAGGCTTTCC

*All probes are complementary to anti-sense strand of the corresponding PCR fragments.

Table S3. Gram stain and isolation source information for the 15 species mixed in mock communities

Species (Strain)	[a]Gram	[b]Source	Reference No.
Bacillus subtilis (NBRC 13719^T)	P	(soil)	4
Bifidobacterium pseudocatenulatum (NBRC 113353)	P	human feces	2
Clostridium butyricum (NBRC 13949^T)	P	Intestine of pig	8
Corynebacterium striatum (NBRC 15291^T)	P	(human nasopharynx)	1
Cutibacterium acnes subsp. *acnes* (NBRC 107605^T)	P	human facial acne	7
Enterocloster clostridioformis (NBRC 113352)	P	human feces	
Lactobacillus delbrueckii (NBRC 3202)	P	(fermented food and feed)	3
Staphylococcus epidermidis (NBRC 100911^T)	P	human nose	10
Streptococcus mutans (NBRC 13955^T)	P	human carious dentine	12
Acinetobacter radioresistens (NBRC 102413^T)	N	cotton sterilyzed by γ–rays	5
Bacteroides uniformis (NBRC 113350)	N	human feces	11
Comamonas terrigena (NBRC 13299^T)	N	hay infusion filtrate	13
Escherichia coli (NBRC 3301)	N	human feces	9
Parabacteroides distasonis (NBRC 113806)	N	human feces	11
Pseudomonas putida (NBRC 14164^T)	N	(soil and water)	6

(a) P and N indicate the Gram-stain positive and negative of each species or strain according to the reference, except for *Enterocloster clostridioformis* , for which we performed Gram staining.

(b) Source indicates the isolation source of each strain according to the NBRC Culture Catalogue (https://www.nite.go.jp/nbrc/catalogue/.), except that Source in parentheses is main habitat or isolation source of each species described in the reference.
